# Amination of Aryl Halides and Esters Using Intensified Continuous Flow Processing

**DOI:** 10.3390/molecules201017860

**Published:** 2015-09-25

**Authors:** Thomas M. Kohl, Christian H. Hornung, John Tsanaktsidis

**Affiliations:** CSIRO Manufacturing Flagship, Bag 10, Clayton South, Victoria 3169, Australia; E-Mails: christian.hornung@csiro.au (C.H.H.); john.tsanaktsidis@csiro.au (J.T.)

**Keywords:** continuous flow processing, flow chemistry, amination, process intensification

## Abstract

Significant process intensification of the amination reactions of aryl halides and esters has been demonstrated using continuous flow processing. Using this technology traditionally difficult amination reactions have been performed safely at elevated temperatures. These reactions were successfully conducted on laboratory scale coil reactor modules with 1 mm internal diameter (ID) and on a preparatory scale tubular reactor with 6 mm ID containing static mixers.

## 1. Introduction

The synthesis of functionalized amine-containing compounds is an area of great interest due to the number of important materials, commodity chemicals and pharmaceutical building blocks relying on this chemistry [1]. There are a number of accepted methods for performing amination reactions, including the Buchwald-Hartwig Reaction [2,3,4,5], Ullmann Reaction [6] and reductive amination [7]. These modern, transition metal-catalysed amination reactions provide excellent control and functional group tolerance, whilst allowing for relatively mild reaction conditions [8]. However, these methods are also expensive and atom inefficient. Classical synthetic routes such as nucleophilic substitution reactions provide a simple and cheap route to many valuable amination products and as such are still used in many industrial processes [9,10,11]. When performing these reactions using traditional batch reactors the temperatures and pressures that may be safely handled become key considerations. This is particularly important when using ammonia or the more volatile amines such as methylamine. As such, batch amination reactions are often performed at room temperature using laboratory glassware, or at elevated temperatures in a pressure vessel such as a sealed tube or a Parr reactor. Scale-up of these processes thus becomes difficult and potentially dangerous [12,13].

Continuous flow processing makes use of tubular and chip-based devices in order to perform chemical reactions [14,15]. The small dimensions of the fluidic pathways result in a well-defined flow regime, high heat and mass transfer rates and a narrow residence time distribution. This allows for excellent control over the reaction process and often improvements in yield, selectivity and product quality [16,17,18]. Continuous flow reactors can also give access to reaction space that can be difficult to access otherwise making it a useful technique for process intensification where temperature and pressure may be a concern in a typical batch reactor [19]. Continuous flow processing has recently been applied to amination reactions using the Buchwald-Hartwig reaction [20,21]. However the use of micro-reactors limits the scalability of these reactions. Additionally, the use of relatively expensive catalysts makes these methodologies unsuitable for many industrial applications. Our work has focussed upon a simple, catalyst free, methodology for the amination of aryl halides and esters. Using continuous flow processing in tubular reactor systems we have demonstrated scalable methods for the intensification of industrially relevant batch processes.

## 2. Results and Discussion

Our initial work focused on the synthesis of 4-amino-3-nitrobenzenesulfonic acid ammonium salt (ANBS) from 4-chloro-3-nitrobenzenesulphonic acid (CNBS). This amination reaction is industrially relevant for the manufacture of the dye Solvent Green 13 (C.I. 10445) and is carried out on a multi-tonne scale [22,23]. It is performed using traditional batch synthesis in a large, sealed, reactor vessel heating under low pressure for several hours. This has several drawbacks, notably the safety issues caused by handling large amounts of pressurised ammonia, lack of control of the reaction exotherm and the relatively poor space-time yield achieved due to long reaction times. Continuous flow processing provides an opportunity to address many of these issues. As only a small amount of reagents are heated at any one time, much higher temperatures and pressures may be used in order to increase the rate of reaction in a safe manner.

**Scheme 1:**
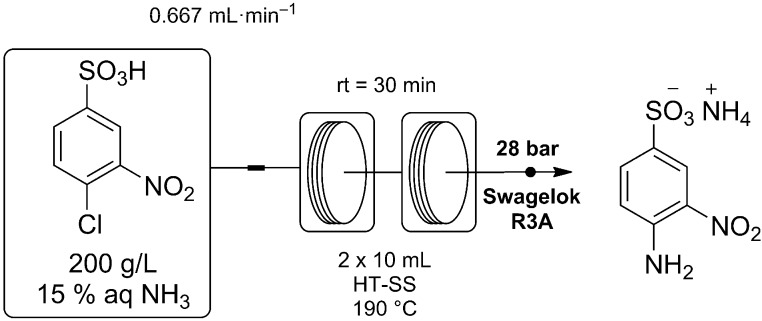
Synthesis of ANBS using a Vapourtec R2/R4 flow reactor for process intensification. The pre-mixed reagent feed was heated to 190 °C with a residence time of 30 min giving complete conversion.

Reactions were performed using a Vapourtec R2/R4 reactor system, fitted with two high temperature stainless steel reactor coils [24]. The reaction was optimised for maximum space-time yield while keeping the system pressure within practical limits. It was found the reaction rate sharply increases with temperature, while the concentration of ammonia has much less of an effect on the rate of conversion for the herein investigated conditions. As such, the concentration of ammonia was reduced to 15 wt % allowing for much higher temperatures to be used due to the reduced vapour pressure. The optimised conditions are shown in Scheme 1 with a 200 g/L stock solution of CNBS prepared in ammonia 15 wt % and pumped through a series of two 10 mL reactor coils at 190 °C with a residence time of 30 min.

Using these conditions a >99% conversion of CNBS was achieved within 30 min, while the industrial batch process takes several hours. Further details of the batch process used industrially have not been provided here due to commercial confidentiality. Workup consists of removal of excess ammonia under reduced pressure followed by concentration of the remaining aqueous phase at an elevated temperature. Upon reaching the point of saturation ANBS may be crystallized and obtained by filtration to give a 96% yield. The space-time yield for this continuous flow reaction, which is defined as the amount of product obtained per hour for one liter of reactor volume, was calculated as 235 g/Lh. In a commercial batch reactor the space time yield is 7.6 g/Lh (based on reaction volume and total processing time) meaning that the flow process gives a 31 fold increase in throughput for a given reactor volume (Table 1, *vide infra*).

The purity of the product is another important consideration, with a low residual chloride content being required for on-processing to the final commercial dye. A sample obtained using our continuous flow processing method was compared with commercially available material (Table 1). Both samples were analysed in duplicate by potentiometric titration. It was found that the commercial sample had an average chloride concentration of 1.74%, while our sample had a chloride content of 0.33%. This represents a 5 fold decrease in residual chloride content using our method.

**Table 1:** Chloride content of 4-amino-3-nitrobenzenesulfonic acid ammonium salt (ANBS) samples obtained through continuous processing and from commercial sources.

Sample	Cl^−^ (%)
ANBS Flow [A]	0.32
ANBS Flow [B]	0.34
Commercial Product [A]	1.74
Commercial Product [B]	1.73

This chemistry has been expanded upon with the synthesis of 4-methylaminopyridine (4-MAP) from 4-bromopyridine hydrochloride. 4-MAP is a useful heterocyclic building block and is a molecule of interest due to its use as a precursor for switchable RAFT agents [25]. Many different literature methods exist for the synthesis of 4-MAP; some are using BuLi with methyl iodide [26] or NaBH_4_ with formaldehyde [27,28] and start from 4-aminopyridine, while others utilize the aryl halide starting material. Most of these methods employ catalysts such as copper to achieve the transformation [29], which are expensive reagents and therefore in many cases prohibitive for large scale chemical manufacturing. The direct synthesis of 4-MAP from aryl halides such as 4-bromopyridine hydrochloride by reaction with aqueous methyl amine is much more desirable and has been performed in batch at elevated temperatures (150–175 °C) and pressures (25 bar) [30,31]. While yields are high (88%), the pressures generated require the use of a sealed tube or Parr reactor and this leads to inherent safety and scale-up issues.

**Scheme 2:**
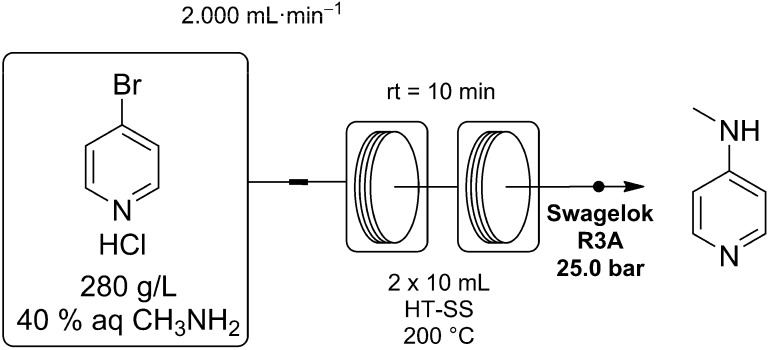
Synthesis of 4-MAP using a Vapourtec R2/R4 flow reactor for process intensification. The pre-mixed reagent feed reacted rapidly at 200 °C, giving the desired product after a residence time of 10 min.

We have now demonstrated that this process may be performed efficiently and safely using a continuous processing method. This reaction was again carried out using a Vapourtec R2/R4 reactor system, fitted with two high temperature stainless steel reactor coils. Here a 280 g/L stock solution of 4-bromopyridine hydrochloride in 40 wt % aqueous CH_3_NH_2_ was passed though the reactor coils, heating the reaction mixture to 200 °C with a residence time of only 10 min (Scheme 2).

A 97% conversion was achieved after this time with subsequent workup and crystallization giving an 80% yield of 4-MAP. The large decrease in processing time results in a much more efficient reaction giving a space-time yield of 768 g/Lh (Table 1, *vide infra*). A comparable result for a batch system, reported by Feng *et al.* [30], equates to a space-time yield of 0.79 g/Lh. As such our process represents a 972 fold increase in the space-time yield over what has been reported using batch processing in the literature.

**Figure 1:**
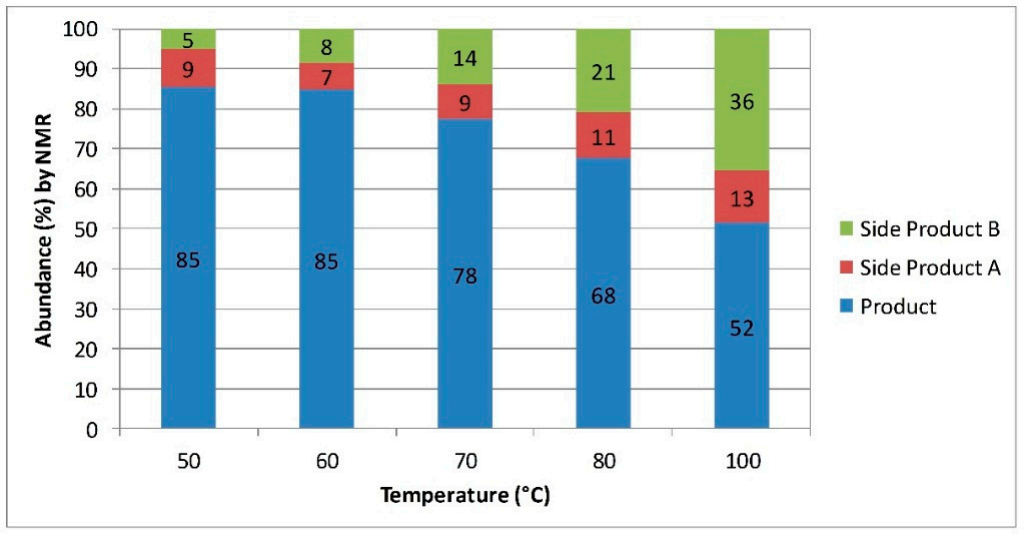
Change in product selectivity with temperature for the synthesis of 3-MPA. Substances A and B are unwanted poly-sulfide side products. These were partially identified by ^1^H-NMR and mass spectrometry, but a comprehensive characterization was not performed.

**Figure 2:**
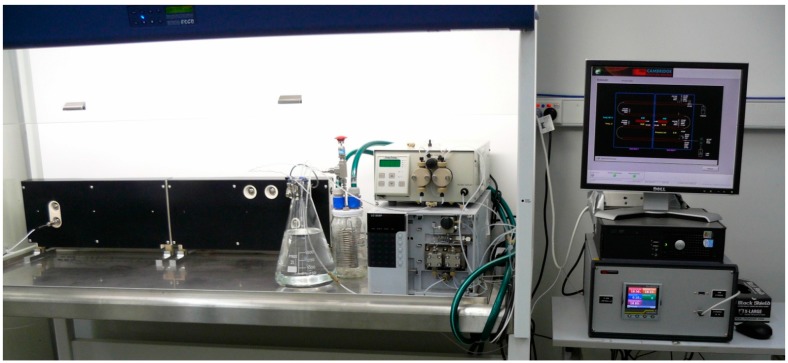
Salamander flow reactor system from Cambridge Reactor Design. This was used for scale-up of the synthesis of 3-MPA. The system consists of the tubular reactor module (left of image), preparative HPLC pumps (image center) and computer-aided process control (right of image).

Furthermore, we have expanded this process to the amination of esters. Here 3-mercaptopropanamide (3-MPA) was targeted as a potential intermediate in the manufacture of water soluble RAFT agents. Once again literature reactions have been found to be relatively inefficient when performed in batch mode, requiring extended processing times in order to achieve acceptable yields [32]. Initial reaction optimization was performed using the Vapourtec system, and it quickly became apparent that high temperatures could not be used for this reaction. Analysis of the product mixture by mass spectroscopy indicated the presence of a mixture of different sulfur-containing compounds. The similarity of these compounds by NMR suggests the formation of a number of polysulfides. These competing reactions become more predominant at higher temperatures leading to a lower 3-MPA yield (Figure 1). Scale-up of this reaction was performed by transferring the reaction conditions developed in the Vapourtec system into a Salamander tubular reactor system from Cambridge Reactor Design (Figure 2) [33].

**Scheme 3:**
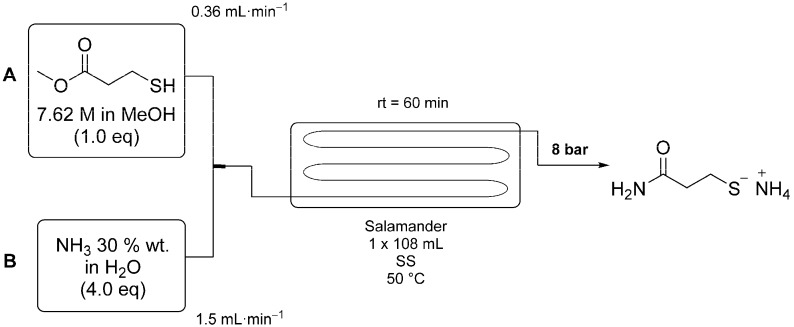
Scale-up of the continuous flow process for the production of 3-MPA using a 100 mL tubular flow reactor from Cambridge Reactor Design. Reagents were fed into the reactor using pumps A (Shimadzu LC-20AP) and B (SSI Prep 100) and mixed in a T-piece. The reaction temperature was set to 50 °C and the residence time to 60 min.

The optimized reaction conditions are shown in Scheme 3. Here methyl 3-mercaptopropionate (1.0 eq) and aqueous ammonia (4.0 eq, 30 wt %) were mixed in a T-piece before passing through the reactor coil, which was heated to 50 °C. The residence time was set to 60 min. Using these conditions a 95% conversion was achieved. The product was not isolated and was on-processed directly as the ammonium salt.

The space-time yield for this reaction may be calculated based on the conversion, giving 131 g/Lh for our continuous process (Table 2). This represents a 60 fold increase in space-time yield over the conversion based result of 2.2 g/Lh from the batch process reported by Gorvin [32].

**Table 2:** Summary of process intensification using continuous flow technology for the synthesis of 4-amino-3-nitrobenzenesulfonic acid ammonium salt (ANBS), 4-methylaminopyridine (4-MAP) and 3-mercaptopropanamide (3-MPA). Conditions for comparable batch reactions are also given.

Entry	Product	Reactor Type	T (°C)	Time (min)	Conversion (%)	Yield (%)	Space-Time Yield (g/Lh)
1	ANBS	Flow	190	30	100	96	235
2		Batch ^a^	n/a	n/a	n/a	n/a	7.6
3	4-MAP	Flow	200	10	97	80	768
4		Batch	175	480	n/a	88	0.79
5	3-MPA	Flow	50	60	95	n/a	131
6		Batch	rt	4200	95	n/a	2.2

^a^ Further details of the batch synthesis of ANBS are not reported due to commercial confidentiality. rt indicates that the reaction was performed at room temperature, with no additional temperature control. n/a indicates that the field does not apply for the reaction listed.

These results demonstrate significant improvements in process intensity for amination reactions of each target. Additionally, the use of continuous flow processing allowed for the safe handling of volatile reagents under high temperature conditions inside the pressurized tubular flow reactors. The aryl halides gave the largest increase in space-time-yield with a 31 fold increase for ANBS and a 972 fold increase for 4-MAP. In both cases much of this increase comes from the ability to conduct the reactions at high temperatures and high pressures in a simple manner using continuous processing technology.

Conducting these reactions using a batch system is limited by the ability to pressurize a traditional batch vessel as well as heat it uniformly. In both cases the reagents are highly activated with the electron-withdrawing nitro group of CNBS and the nitrogen of the pyridine ring leading to more mobile halogen leaving groups. The need for such groups has been well documented in the past and is one of the factors limiting this methodology from becoming a general approach for producing aryl amines. However, we have shown that where such substrates may be used direct amination is a viable, scalable option when using continuous flow processing.

The amination of esters gave more modest improvements in efficiency. Here continuous flow processing could again be used to conveniently heat aqueous ammonia 30 wt % in a pressurized environment. This resulted in a 60 fold increase in space-time-yield over the batch system. Further improvement in the processing time was limited by side reactions occurring between the thiol functionality of the substrate. These side reactions became more prevalent at higher temperatures resulting in a lower abundance of the desired 3-MPA. By tuning the temperature we were able to reach a set of conditions providing improvements in reaction efficiency while minimizing the amount of size product formed.

## 3. Experimental Section

### 3.1. Materials and Analysis

The reagents methylamine (40 wt %) and methyl 3-mercaptopropionate were obtained from Sigma-Aldrich (Castle Hill, Australia); 4-chloro-3-nitrobenzenesulfonic acid sodium salt (67% purity) was obtained from Prashant Industries (Gurjarat, India); ammonia (30 wt %) from Chem-Supply (Gillman, Australia) and 4-bromopyridine hydrochloride (>97%) from Boron Molecular (Melbourne, Australia). The solvents isopropanol (*i*-PrOH) and dichloromethane (CH_2_Cl_2_) were obtained from Merck (Darmstadt, Germany) and methanol (MeOH) was obtained from Sigma-Aldrich. All reagents and solvents were used without further purification.

Reaction conversions were calculated from ^1^H-NMR spectra, which were recorded on an AC-400 spectrometer (Bruker, Preston, Australia) in deuterated water or DMSO (from Cambridge Isotope Laboratories Inc., Tewksbury, MA, USA). The residual solvent peak at δ = 7.26 ppm was used as an internal reference. Product compositions were confirmed by GC-FID and GC-MS. GC-mass spectra were obtained with a Clarus 600 GC mass spectrometer (Perkin Elmer, Melbourne, Australia) using electron impact ionization in the positive ion mode with an ionization energy of 70 eV. The gas chromatography was performed with a Perkin Elmer Elite-5MS GC column (30 m × 0.25 mm ID, 0.25 μm film thickness), with a temperature program of 40 °C for 2 min, then heating at 10 °C/min to 280 °C where the temperature was held for 4 min with a split ratio of 70, an injector temperature of 250 °C and the transfer line set to 250 °C. Ultra high purity helium was used as the carrier gas with a flow rate of 0.7 mL/min. GC-FID analysis were performed on a 6850 Series II gas chromatograph (Agilent, Mulgrave, Australia) with a split/splitless inlet and a detector temperature of 250 °C. Separation was done on a BPX5 capillary column (Grace, Epping, Australia, 25 m × 0.32 mm ID, 0.50 μm film thickness), with a temperature program of 40 °C for 2 min, then heating at 10 °C/min to 280 °C where the temperature was held for 4 min with a split ratio of 50 and an injector temperature of 200 °C. High purity helium was used as the carrier gas with a flow rate of 2.4 mL/min.

### 3.2. Experimental Configuration of the Vapourtec Flow Reactor

The Vapourtec R2/R4 system (Vapourtec Ltd, Suffolk, UK) was fitted with two, 10 mL, 316L SS, high temperature reactor modules. These modules may be operated in temperatures ranging from ambient to 250 °C. The pumping unit was fitted with two, standard, Vapourtec HPLC pumps capable of delivering flow rates from 0.010 to 9.99 mL/min at reaction pressures of up to 42 bar. System pressure was maintained using a Swagelok R3A-B relief valve, which can be adjusted to give system pressures ranging from 24.1 to 51.7 bar. All other plumbing of reactor lines was carried out using 316 SS tubing (1/16″ OD, 0.040″ ID) obtained from VICI (Houston, TX, USA) and the appropriate SS fittings from Swagelok (Campbellfield, Australia). Where required a custom made cooling coil was used in order bring the product stream to ambient temperature prior to collection. This was constructed from a 6 m length of coiled SS tubing (1/8″ OD, 0.028″ ID) submerged in a glass housing through which water could flow. In cases where air cooling was sufficient a 1 m coil of the above tubing was instead used prior to collection.

### 3.3. Experimental Configuration of Salamander Flow Reactor

The Salamander tubular reactor system (Cambridge Reactor Design, Cottenham, UK) consists of a series of SS (8 mm OD, 6 mm ID) tubes connected in a serpentine fashion within the reactor housing. The reactor is fitted with static mixers along its length and has a total internal volume of 108 mL. The system is electrically heated giving an operating temperature range from 20 °C to 150 °C and may hold a pressure of up to 30 bar. Heating is controlled by the Continuous Reactor System (CRS) software application, which also provides monitoring of the internal temperature and pressure. System pressure was maintained using a Swagelok R3A-A relief valve, which can be adjusted to give system pressures ranging from 3.4 to 24.1 bar. All other plumbing of reactor lines was carried out using Swagelok fittings. Optional cooling modules may be added as described for the Vapourtec system. Two pumps were plumbed into the system. The first is a LC-20AP preparative HPLC pump (Shimadzu, Rowville, Australia) capable of flow rates of up to 100 mL/min (up to 420 bar) and 150 mL/min (up to 300 bar). The second is a Scientific Systems Incorporated (SSI, State College, PA, USA) Prep 100 Pump (P40PFXP1) capable of delivering flow rates from 0.1 to 100 mL/min at pressures of up to 276 bar.

### 3.4. Synthesis of 4-Amino-3-nitrobenzenesulfonic Acid Ammonium Salt (ANBS)

CNBS (40.00 g, 67% purity) was dissolved in aqueous ammonia solution (15 wt %) sufficient to give a total volume of 200 mL. The resulting 200 g/L stock solution was then pumped at a rate of 0.667 mL/min through two 10 mL, high temperature, stainless steel, reactor coils and heated to 190 °C. The combined reagents were directed through a 1 m, stainless steel, cooling coil, prior to passing through the adjustable back pressure regulator set to maintain 28 bar. The orange product solution was collected at the reactor outlet over the course of 4 h and 4 min, with analysis of the crude solution indicating 100% conversion of the starting material. The total amount of CNBS processed for collection was 21.78 g (91.7 mmol). The excess NH_3_ was then removed under vacuum and the product solution concentrated to allow crystallisation of the product. The resulting orange crystals were then collected by filtration, washing first with cold water then with iPrOH. The crystals were then dried in the oven at 100 °C and ground to give a total of 19.11 g ANBS as a fine yellow powder in 96% yield (3 crops). ^1^H-NMR (D_2_O, 400 MHz) δ 6.86 (d, *J* = 9 Hz 1H, Ar-H), 7.55 (dd, *J* = 9, 2 Hz, 1H, Ar-H), 8.30 (d, *J* = 2 Hz, 1H, Ar-H); ^13^C-NMR (D_2_O, 100 MHz) δ 119.6, 123.5, 129.0, 129.9, 131.9, 147.0. Samples were analysed for chloride concentration using a MPT 798 Titrino potentiometric titrator (Metrohm, Gladesville, Australia). The samples were dissolved in water with a small amount of nitric acid and titrated directly for chloride via auto titration. Duplicate analysis was performed on all samples as shown with [A] and [B].

### 3.5. Synthesis of 4-Methylaminopyridine (4-MAP)

4-Bromopyridine hydrochloride (1.40 g, 7.2 mmol) was dissolved in cooled, aqueous methylamine (40 wt %) sufficient to give a total volume of 5 mL. The resulting 1.45 M stock solution was then pumped at a rate of 2 mL/min through two 10 mL, high temperature, stainless steel, reactor coils and heated to 200 °C. The reagent stream was directed through a 6 m stainless steel cooling coil, prior to passing through the adjustable back pressure regulator set to maintain 25 bar. The light yellow product solution was collected at the reactor outlet before extraction with CH_2_Cl_2_ (5 × 10 mL). The combined organic layers were dried over MgSO_4_, filtered and the solvent evaporated to give 0.62 g of 4-methylaminopyridine as a white crystalline solid in 79% yield. ^1^H-NMR (D_2_O, 400 MHz) δ 2.67 (s, 3 H, CH_3_), 6.49 (d, *J* = 8 Hz, 2H, Ar-H), 7.93 (d, *J* = 8 Hz, 2H, Ar-H); ^13^C-NMR (D_2_O, 100MHz) δ 28.4, 107.6, 148.1, 156.0.

The above procedure has also been used to process 4-BPH (467.60 g) as a 1.67 L (1.45 M) stock solution in aqueous methylamine (40 wt %) over the course of 14 h. After workup 213.7 g of 4-MAP was obtained in 80% yield and 97% purity by GC-FID.

### 3.6. Synthesis of 3-Mercaptopropanamide (3-MPA)

Methanol (1.85 mL) was added to methyl 3-mercaptopropionate (10.00 mL) in order to give a 7.62 M stock solution. This was pumped at 0.133 mL/min, mixing in a T-piece with a stream of aqueous ammonia (30 wt %) pumped at 0.533 mL/min. The combined reagents were directed through four 10 mL, PFA, reactor coils and heated to 50 °C with a residence time of 60 min. The clear product solution was collected for 1 h 42 min giving 67.9 mL, with a conversion of 95% indicated by ^1^H-NMR (D_2_O). The excess NH_3_ was then removed under vacuum and the wet product dried overnight in a vacuum desiccator to give 3-mercaptopropanamide (7.42 g) as a white solid in 68% yield (87% purity). ^1^H-NMR (DMSO, 400 MHz) δ 2.31 (t, *J* = 7 Hz, 2H, CH_2_), 2.58 (t, *J* = 7 Hz, 2H, CH_2_), 6.82 (s, 1H, NH), 7.30 (s, 1H, NH); ^13^C-NMR (DMSO, 100 MHz) δ 20.4, 39.6, 172.9.

The above procedure has also been performed on a larger scale using an electrically heated Salamander (108 mL internal volume) flow reactor. Methyl 3-mercaptopropionate (50.6 mL, 7.6 M) was pumped at 0.36 mL/min and aqueous ammonia (4.0 eq, 30 wt %) pumped at 1.5 mL/min. The reagent streams were mixed in a T-piece before passing though the heated (50 °C) reactor with a residence time of 60 min. The product solution was collected into a 1 L round bottom flask over the course of 3.5 h with 95% conversion of the starting material confirmed by ^1^H-NMR (D_2_O) and a product purity of 90%.

## 4. Conclusions

In summary, we have demonstrated significant improvements in process intensity for amination reactions of aryl halides and esters. This was achieved by using continuous flow processing, allowing for the safe handling of volatile reagents under high temperature conditions inside pressurized tubular flow reactors. Scale up of this process was also demonstrated using a 100 mL continuous flow reactor with direct transfer of conditions. This simple metal-free amination methodology can be applied where sufficiently active substrates are used and where pressure to minimize cost makes many catalytic methods unviable. In many cases this reaction can be performed solvent free with the aryl halide or ester pre-mixed with the amine source, this has both environmental and cost benefits. We have focused on processes which may be of industrial interest, such as the amination of CNBS to produce the dye precursor ANBS. There is much potential for expanding the scope of this work to include different substrates making use of the ability of continuous flow processing to safely handle volatile reagents at elevated temperatures.
